# Why It Is Important to Develop an Effective and Safe Pediatric COVID-19 Vaccine

**DOI:** 10.3390/vaccines9020127

**Published:** 2021-02-05

**Authors:** Nicola Principi, Susanna Esposito

**Affiliations:** 1 Università degli Studi di Milano, 20122 Milan, Italy; nicola.principi@unimi.it; 2 Pediatric Clinic, Pietro Barilla Children’s Hospital, University of Parma, 43126 Parma, Italy

**Keywords:** children, COVID-19, pediatric infectious diseases, SARS-CoV-2, vaccine

## Abstract

The need to cope with the medical, social, and economic storm due to the new coronavirus 2019 (COVID-19) pandemic as quickly as possible has led to the very rapid development of a huge number of vaccines. All these vaccines have been mainly developed in healthy adults and, in some cases, in the elderly. Children were marginally involved as, according to the clinical trial registry Clinical Trials.gov, only very few studies have included children among subjects to enroll, although just a few weeks after the pandemic declaration, the US Food and Drug Administration had highlighted the importance of vaccine evaluation in pediatrics. Availability of an effective and safe pediatric COVID-19 vaccine appears mandatory for several clinical and epidemiological reasons. However, as the development of an effective and safe pediatric vaccine seems far from easy, strong cooperation among governments, researchers, and pharmaceutical companies is highly desirable.

## 1. Background

The need to cope with the medical, social, and economic storm due to the new coronavirus 2019 (COVID-19) pandemic as quickly as possible has led to the very rapid development of a huge number of vaccines, in some cases using novel development-and-manufacturing platforms that are highly adaptable and speed up development considerably. For COVID-19, several viral vectors and nucleic acid-based vaccines are in clinical development. [1]. Some of these have been licensed for clinical use even before a year has passed since the pandemic declaration. All these vaccines have been mainly developed in healthy adults and, in some cases, in the elderly. Children were marginally involved [2] despite just a few weeks after the evidence of the pandemic disaster, the US Food and Drug Administration had reported in the Guidance to assist sponsors in the clinical development and licensure of vaccines against COVID-19 that was important to plan for assessments of safety and effectiveness of these preparations in pediatrics [3].

## 2. Trials on COVID-19 Vaccines in Children

Only a few trials have included children among patients to enroll. One of them, a phase 2/3 trial of an mRNA vaccine (NCT04368728), was first posted on ClinicalTrials.gov. on 30 April 2020 and has an estimated primary completion date of 30 July 2021. Two other trials regarding an inactivated vaccine (NCT04551547) and an mRNA vaccine (NCT04649151) are phase 1/2 and phase 2/3 trials and were first posted on 16 September 2020 and 2 December 2020, respectively. However, the first has an estimation study completion date on September 2021 and the second is not yet recruiting. In all the cases, the number of children involved is relatively small, and the results are expected only in several months. The delay in the development of COVID-19 vaccines in pediatrics retraces what regularly occurs for all pharmaceutical products. Children and pregnant women are routinely excluded from the initial trials for fear of a greater risk of serious adverse events than in healthy adults. Moreover, in the case of the COVID-19 pandemic, the urgency for a pediatric vaccine was not recognized, given the lower susceptibility of children to the infection and the greater prevalence of asymptomatic or poorly symptomatic cases compared to adults and the elderly [4]. Children were not considered among groups for early vaccination if vaccine supply was limited, contrarily to healthcare personnel, workers in essential and critical industries, people at high risk for severe COVID-19 illness due to underlying medical conditions, and people 65 years and older [5]. However, a more in-depth analysis of the impact of severe acute respiratory syndrome coronavirus 2 (SARS-CoV-2) infection in pediatrics might reverse this evaluation.

## 3. Why It Is Important to Develop a Pediatric COVID-19 Vaccine

Pediatric COVID-19 has morbidity and mortality characteristics quite like those of several pediatric infectious diseases, including influenza, for which effective vaccines are available and are strongly recommended worldwide. Although rarely, pediatric COVID 19 can be as severe as influenza and lead to death in a similar number of children. A study carried out in school-age children with laboratory-confirmed COVID-19 or influenza revealed that hospitalization rate, intensive care unit (ICU) admission rate, and use of mechanical ventilators were quite similar in both groups [6]. The need for hospitalization was documented in 17% vs. 21% of the cases, admission to the ICU in 6% vs. 7%, and use of ventilators in 3% vs. 2%, respectively. In the USA, from the beginning of the pandemic to 1 October 2020, a total of 112 pediatric death COVID-19 cases have been reported [7], a number not dissimilar to that due to influenza in each of the most recent influenza seasons. On the other hand, the relative clinical importance of pediatric COVID-19 cannot be considered a limit to developing a pediatric COVID-19 vaccine. On the other hand, vaccines against varicella, rubella, rotavirus, and hepatitis A were recommended despite these diseases had, before vaccine introduction, mortality rates similar to that presently found for COVID-19 [8].

The clinical impact of an effective and safe COVID-19 vaccine could be sufficient to develop the vaccine and to recommend it in children. However, other factors can further justify the production as fast as possible of a COVID-19 vaccine for children. Use of influenza vaccine in children, even in those otherwise healthy that generally have a mild disease, has been associated with a reduced risk of influenza in the elderly with enormous medical, social, and economic advantages [9]. It could be hypothesized that similar advantages could be obtained with an effective COVID-19 vaccine. Children with SARS-CoV-2 infection, even when asymptomatic, carry the virus in the nasopharynx for several days and can potentially spread the virus. How much younger children play a role in this regard is presently unknown [10], but it is documented that teenagers may spread the virus and contribute to the disease diffusion. A study carried out in South Korea in which 59,073 contacts of 5706 COVID-19 index patients were monitored for an average of 9.9 days after the detection of SARS-CoV-2 infection showed that the COVID-19 positivity rate for contacts of older children (10–19 years) exceeded 18%, compared to 5.3% for contacts of younger children (0–9 years), with an overall study positivity rate of 11.8% [11]. This study has some limitations as the number of infections might have been underestimated because all asymptomatic patients might not have been identified, and detected cases could have resulted from exposure outside the household. However, the importance of older children and adolescents as a cause of SARS-CoV-2 transmission seems confirmed by the epidemiology of COVD-19 after the lockdown ended. This summer, a relevant increase in the number of COVID-19 cases starting at pubs, nightclubs, and discos was evidenced in several countries. Teenagers and young adults were the main frequenters of these clubs and usually paid little attention to compliance with the rules for infection prevention [12].

Finally, it seems possible that vaccine prevention of COVID-19 in children could reduce the indirect consequence of COVID-19. As occurs with other vaccines, pediatric vaccination can significantly impact the total number of SARS-CoV-2 infections. What has been demonstrate for the pneumococcal conjugate vaccines that has been found effective in reducing incidence of pneumococcal infections in unvaccinated adults and old people, is a good example in this regard [13]. Reduction in total COVID-19 cases can limit disruptions to health services, with a high impact on child health and risk of reversing decades of progress toward eliminating preventable child deaths. A recent World Health Organization (WHO) survey in over 100 countries showed that disruptions in services mainly devoted to noncommunicable disease diagnosis and treatment, treatment for mental health disorders, and cancer diagnosis and treatment were evidenced in 69%, 61%, and 55% of the studied countries, respectively [14].

The need for giving decisive acceleration to the development of a COVID-19 pediatric vaccine is further suggested by the evidence that the identification of an effective and safe pediatric vaccine generally takes much more time than the development of a vaccine for adults. Enrollment of children in a clinical trial requires the informed consent of parents or legal guardian and, when the children are more than 8 years old, of the children themselves. This can be a limit to a rapid achievement of the number of cases to enroll. Moreover, the immune system response to antigen stimulation varies significantly during childhood [15]. This means that the antigen content of each vaccine dose and the number of doses capable of evoking a protective immune response can be different in infants and toddlers compared to school-age children and adolescents and that numerous clinical trials for each pediatric age must be performed before all children can be adequately vaccinated. Moreover, the need for booster doses can be different, and this leads to specific monitoring of persistence of protection. Specific monitoring is also required to obtain reliable data on tolerability and safety, taking into account that, as reported for other pediatric vaccines, some severe adverse events can be relatively uncommon and are identified only after tens of thousands of doses have been administered. The example of the rotavirus vaccine and the development of intussusception is paradigmatic in this regard [16]. Furthermore, particular attention must be paid to the problems of the immune response evoked by the COVID-19 vaccines in pediatrics. Due to the potential different immune response of children compared to adults, the risk of enhanced respiratory disease (ERD) or antibody-dependent enhancement of COVID-19 severity after vaccine administration must be carefully considered before pediatric vaccines are licensed. ERD in children receiving a formalin-inactivated, alum adjuvanted, whole-virus, respiratory syncytial virus (RSV) vaccine was reported more than 50 years ago [17]. Induction of antibodies with poor neutralizing activity which favor virus penetration and/or Th2 cell responses associated with severe inflammation was considered the main reason for this phenomenon. As vaccines in development were found to evoke neutralizing antibodies and a Th1 response, this immune abnormality seems averted. Results of a study regarding an inactivated whole-virus COVID-19 vaccine that was immunogenic and without relevant adverse events are in line with this supposition [18]. However, the evidence that children with COVID-19 and the multisystem inflammatory syndrome in children (MIS-C) have significantly higher serum antibody levels to SARS-CoV-2 than asymptomatic children and patients with mild disease [19] suggests that antibodies to SARS-CoV-2 can play a significant role in conditioning MIS-C development.

## 4. Conclusions

Availability of an effective and safe pediatric COVID-19 vaccine appears mandatory for several clinical and epidemiological reasons. However, as the overcoming of all the problems that may delay the achievement of this goal is very difficult, strong cooperation among governments, researchers, and pharmaceutical companies is highly desirable.

## Data Availability

Not applicable for a Viewpoint.
